# Human cardiovascular disease model predicts xanthine oxidase inhibitor cardiovascular risk

**DOI:** 10.1371/journal.pone.0291330

**Published:** 2023-09-08

**Authors:** Ryan E. Feaver, M. Scott Bowers, Banumathi K. Cole, Steve Hoang, Mark J. Lawson, Justin Taylor, Brian D. LaMoreaux, Lin Zhao, Brad R. Henke, Brian A. Johns, Andrew C. Nyborg, Brian R. Wamhoff, Robert A. Figler

**Affiliations:** 1 HemoShear Therapeutics, Incorporated., Charlottesville, Virginia, United States of America; 2 Horizon Therapeutics plc, Deerfield, Illinois, United States of America; Helwan University, EGYPT

## Abstract

Some health concerns are often not identified until late into clinical development of drugs, which can place participants and patients at significant risk. For example, the United States Food and Drug Administration (FDA) labeled the xanthine oxidase inhibitor febuxostat with a”boxed” warning regarding an increased risk of cardiovascular death, and this safety risk was only identified during Phase 3b clinical trials after its approval. Thus, better preclinical assessment of drug efficacy and safety are needed to accurately evaluate candidate drug risk earlier in discovery and development. This study explored whether an *in vitro* vascular model incorporating human vascular cells and hemodynamics could be used to differentiate the potential cardiovascular risk associated with molecules that have similar on-target mechanisms of action. We compared the transcriptomic responses induced by febuxostat and other xanthine oxidase inhibitors to a database of 111 different compounds profiled in the human vascular model. Of the 111 compounds in the database, 107 are clinical-stage and 33 are FDA-labelled for increased cardiovascular risk. Febuxostat induces pathway-level regulation that has high similarity to the set of drugs FDA-labelled for increased cardiovascular risk. These results were replicated with a febuxostat analog, but not another structurally distinct xanthine oxidase inhibitor that does not confer cardiovascular risk. Together, these data suggest that the FDA warning for febuxostat stems from the chemical structure of the medication itself, rather than the target, xanthine oxidase. Importantly, these data indicate that cardiovascular risk can be evaluated in this *in vitro* human vascular model, which may facilitate understanding the drug candidate safety profile earlier in discovery and development.

## Introduction

A 2016 meta-analysis revealed that 90% of compounds failed in phase 2/3 clinical trials, 30% due to safety concerns, reflecting high failure rate in the drug discovery process [1]. A $1–2 billion cost and 10–15 year timeline per drug to be approved for clinical use exhibits the overwhelming financial and time burden, suggesting the need for more efficient tools that can be used earlier during drug optimization [2]. Here, we describe a predictive model that combines biological responses of human tissues with pathway analyses that could help triage problematic compounds and mechanisms earlier in drug development.

Numerous strategies are commonly deployed in drug development for de-risking pre-clinical molecules. *In vitro* models have advantages over more traditional *in vivo* safety and efficacy studies, such as decreased ethical considerations, increased experimental control and throughput. However, *in vitro* models often lack physiological relevance bringing into question the translatability of data obtained. A strategy we have taken is to design advanced human *in vitro* systems that restore the physiological microenvironment and cellular responsiveness of multicellular tissues across several disease states, such as propionic acidemia [3–6], cancer [7], liver diseases [8,9] including non-alcoholic steatohepatitis [10–13], and vascular diseases [14–17] including atherosclerosis [16–21]. These advanced model systems can be coupled with robust transcriptional profiling across a broad spectrum of experimental treatments. Compiling these data into a human response database is particularly useful in understanding complex or understudied biological responses.

In this study, we sought to test the utility of a human drug-response database for understanding cardiovascular safety of front-line gout medications. These medications have a well-developed clinical profile, yet recent clinical trial data appears to contradict United States Food and Drug Administration (FDA) warnings of cardiovascular risk.

**Fig 1:**
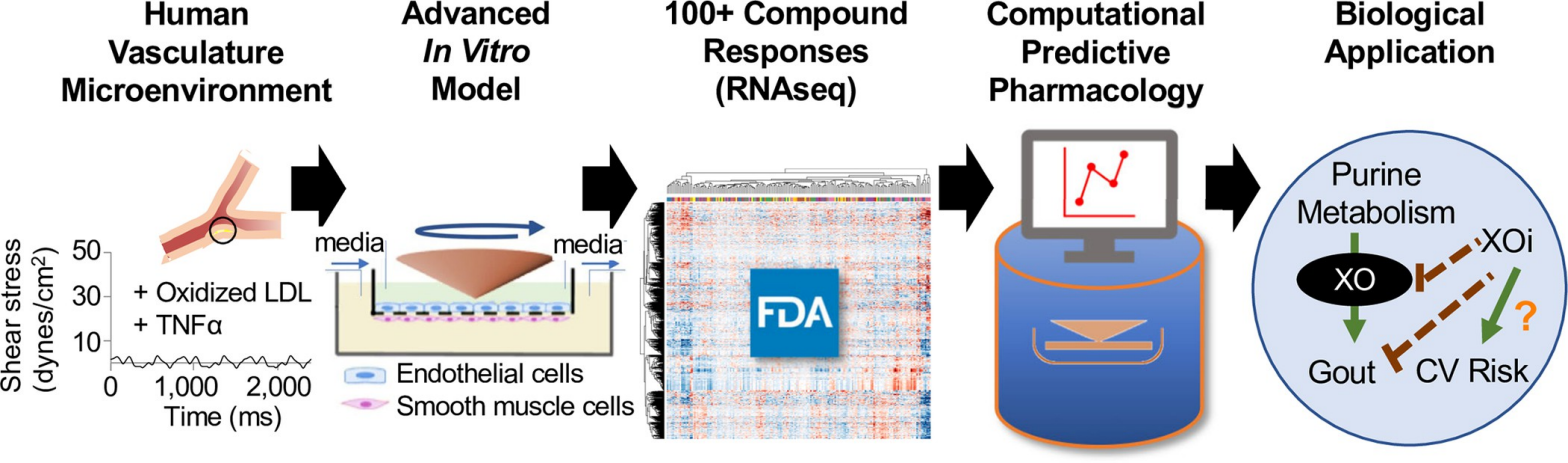
Study overview. Schematic representing the experimental pipeline of an advanced *in vitro* human vascular surrogate model to create a response database from FDA-regulated compounds. Computational biology tools identified pharmacological classifiers that predicted cardiovascular risk. This database was applied to xanthine oxidase (XO) inhibitors, such as febuxostat, to explore responses and provide context to apparently contradictory clinical trial results.

Gout is the most common inflammatory arthritis, and the frequent presence of specific comorbidities, including cardiovascular disease and chronic kidney disease increase the mortality rate. In addition to anti-inflammatory therapies for flares, gout is primarily managed with the xanthine oxidase inhibitors febuxostat or allopurinol to lower serum urate levels. Febuxostat is widely prescribed as a second-line therapy where allopurinol is ineffective, not tolerated, or contraindicated [22–24] and can be used as a first-line therapy for kidney-impaired gout patients (~30–50% of affected individuals). However, the FDA mandated febuxostat carry its most prominent warning due to all-cause cardiovascular-related death identified in a large, manufacturer-sponsored clinical trial (CARES, NCT01101035) [25]. Increased cardiovascular risk has not been reported with allopurinol, and it is unknown how febuxostat mediated cardiovascular risk in the CARES trial. However, a more recent febuxostat trial (FAST, EudraCT 2011-001883-23) [26] in patients without pre-existing cardiovascular risk refutes this designation [27]. Thus, we sought to evaluate the utility of an advanced vascular model to detect the potential of xanthine oxidase inhibitors to mediate cardiovascular risk (**Fig 1**).

## Materials and methods

### Cell culture

Primary human endothelial cells (ECs) and smooth muscles cells (SMCs) of aortic origin were purchased from Lonza (USA). ECs and SMCs were maintained in EGM-2 BulletKit (Lonza CC-3162) and SmGM-2 BulletKit (Lonza CC-3182) media, respectively.

### Human-derived LDL

Human native low-density lipoprotein LDL (nLDL) was purchased from Kalen Biomedical (770200) and oxidized via the following procedure: nLDL was dialyzed overnight in PBS to remove EDTA, then dialyzed in PBS containing 13.8 μM CuSO_4_ for three days. Next, the oxidized LDL (oxLDL) was dialyzed in PBS containing 50 μM EDTA to remove excess copper. The oxidative state of LDL was then confirmed via electrophoretic migration of oxLDL versus nLDL [16].

### Transwell co-culture plating conditions and hemodynamic exposure

**Fig 2:**
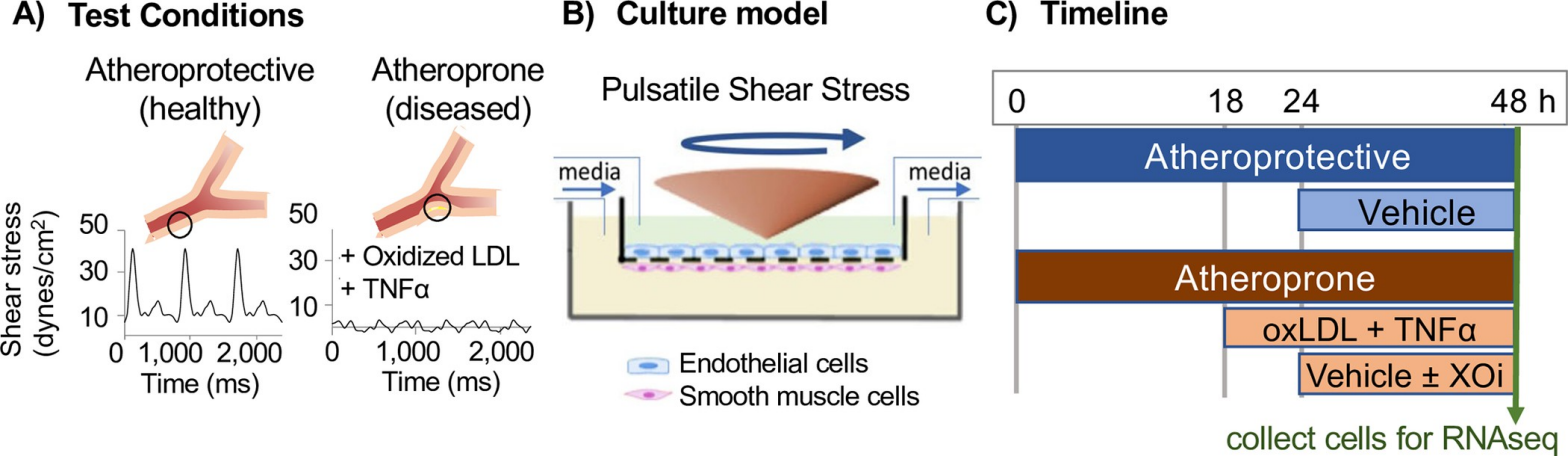
*In vitro* advanced vascular model. **A)** Human hemodynamic shear stress acquired from magnetic resonance imaging of atheroprotective and atheroprone vascular beds. **B)** Human primary vascular endothelial and smooth muscle cells were co-cultured under atheroprone shear stress, pro-inflammatory oxidized low-density lipoprotein (oxLDL, 50 μg/ml), and tumor necrosis factor alpha (TNFα, 50 pg/ml) to mimic the atheroprone state. **C)** Experimental timeline. After experimentation, RNA-seq was performed on each cell type.

The hemodynamic device with the transwell co-culture plating was previously detailed [17] and illustrated in **Fig 2B**. Briefly, the top and bottom surface of a porous polycarbonate transwell membrane (Corning, Inc.) was coated with 0.1% gelatin. First, human SMCs were plated on the bottom transwell surface, and then human ECs were plated on the top transwell surface two days later. Transwells with cells were then placed in fresh media (M199 supplemented with 2% FBS, 2 mM L-glutamine, and 100 U/ml penicillin-streptomycin) until shear stresses were applied the next day.

Human hemodynamic shear stress profiles were derived from magnetic resonance imaging of the human common carotid artery and internal carotid sinus, areas of healthy, atheroprotective regions, or areas prone to atherosclerosis (atheroprone), respectively [18,28] (**Fig 2A and 2C**). Wall shear stress values were calculated from the blood velocity profiles and applied in the EC layer of the co-culture transwell using a direct drive cone-and-plate device (**Fig 2A**) [17,18]. Dextran (4%) was included in the media to obtain the viscosity necessary for transmitting waveform-specific shear forces from the motor/cone assembly to the EC layer of the transwell setup.

### Normal and pathological hemodynamic conditions and drug treatments

Cells were primed with atheroprotective or atheroprone hemodynamics for 18 hours, and then exposed to physiologically relevant concentrations of human oxidized low-density lipoprotein (oxLDL; 50 μg/ml) and human recombinant tumor necrosis factor-α (TNFα; 0.05 ng/ml; R&D Systems 210-TA-010) in the atheroprone condition for an additional 6 hours prior to test compound addition (**Fig 2C**).

111 test compounds were initially selected to span a diverse range of mechanisms, structures, and known clinical outcomes. Following priming, cells were exposed to a vehicle control (0.1% dimethyl sulfoxide, DMSO) or compounds at concentrations approximating Cmax (based on human PK at therapeutic doses), or 10x Cmax, for 24 hours (**Fig 2C**). All experiments were terminated after 48 hours and cells harvested for RNA-seq studies and data used to construct that human response database.

Similarly, xanthine oxidase inhibitors were evaluated after atheroprone priming: febuxostat (FBX at 10, 100 μM), the allopurinol active metabolite oxypurinol (OXP at 50, 500 μM), topiroxostat (TPS at 10, 100 μM), and a structural analog of febuxostat (FBX-1 at 10, 100 μM). For xanthine oxidase inhibitors, minimal to no effects were observed for compounds dosed at lower concentrations, and thus 10x concentration data is presented.

### RNA preparation and RNA sequencing

After 48 hours of test compound exposure under atheroprone conditions, RNA was separately harvested from ECs and SMCs using the Invitrogen Purelink RNA Mini kit (12183018A) and RNA concentration determined with the Nanodrop according to manufacturer’s instructions. 250 ng RNA per sample was submitted to Expression Analysis, Inc. for Illumina-based RNA deep sequencing and approximately 20 million, 50 base pair, paired-end reads were generated per sample. For each treatment, 4 EC and 4 SMC samples were run per experiment.

### Data analysis and statistics

#### Differential expression analysis

Following RNA sequencing, reads were aligned to the human transcriptome build GRCh38, version 91 using the Salmon RNA-seq quantification tool [29]. Gene-level expression quantification was performed using the ‘tximport’ Bioconductor package [30]. Differential expression analysis was performed on these values using the ‘edgeR’ Bioconductor package [31]. This analysis was independently performed on ECs and SMCs.

#### Comparison to our human drug response database

The human response RNA-seq data for 111 compounds in the human hemodynamic co-culture model underlying this study were created as a proprietary dataset by HemoShear Therapeutics. Together, these compound responses constitute a human drug-response database (HRDB) of transcriptomic data generated in the same experimental system across 111 compounds tested in the system. Of those 111 compounds, 107 are clinical stage compounds with 33 carrying FDA boxed warnings for cardiovascular risk. The full list of compounds and any information regarding their boxed warning for cardiovascular risk can be found in S1 Table. Side effects and adverse events for these compounds obtained through SIDERDB [32] and OFFSIDES [33] were used to create side effect prediction models. The treatments were binned as either negative or positive for a given side effect based on clinical outcomes and a prediction model was trained and tested for each one using 10-fold cross-validation. These prediction models consisted of combinations of feature selection methods and classification algorithms, with the best performing model chosen based on highest mean AUC (area under the receiver operating characteristic, ROC, curve) value based on 150 bootstrap resamplings to the training / test data. The classification algorithms were implemented using caret [34] and consisted of lasso, ridge regression, support vector machines with linear kernel, support vector machines with radial basis function kernel, elasticnet, random forest, and knn. Feature selection methods consisted of filtering down the total gene set to those that were significantly differentially expressed when compared to the non-treatment atheroprone control in at least a subset of the training data and using both fold change data from this comparison as well as transcripts per million (TPM) expression values.

We made functional comparisons to test compound treatments in the database by comparing the log fold changes of genes across Reactome pathways. For a given Reactome pathway, the Spearman correlation of log fold changes was calculated between the treatments in this study and the treatments in the HRDB. Only genes that had treatment p-value < 0.05, in either of the two contrasts being compared, were considered for analysis. Correlation values were calculated across all elements of the Cartesian product of treatments in the HRDB, the xanthine oxidase inhibitor treatments in this study, and all Reactome pathways. The resulting set of correlation values allowed us to identify pathways where the xanthine oxidase inhibitor treatments had an exceptionally high degree of similarity with HRDB drugs that are associated with cardiovascular risk. This assessment was made by identifying pathway-drug combinations where cardiovascular-risk drugs tended to have a higher correlation than drugs without known cardiovascular risk. This identification was performed with the Mann-Whitney U-test. In addition, Fisher’s exact test was used to assess the enrichment of cardiovascular-risk drugs among drugs with positive pathway-level correlation. The ultimate result of this analysis was a list of pathways for each xanthine oxidase inhibitor, where each pathway is similarly regulated by the xanthine oxidase inhibitor and the set of cardiovascular-risk drugs in the HRDB. All data and code are available at DOI: 10.5281/zenodo.8144264.

## Results

**Fig 3:**
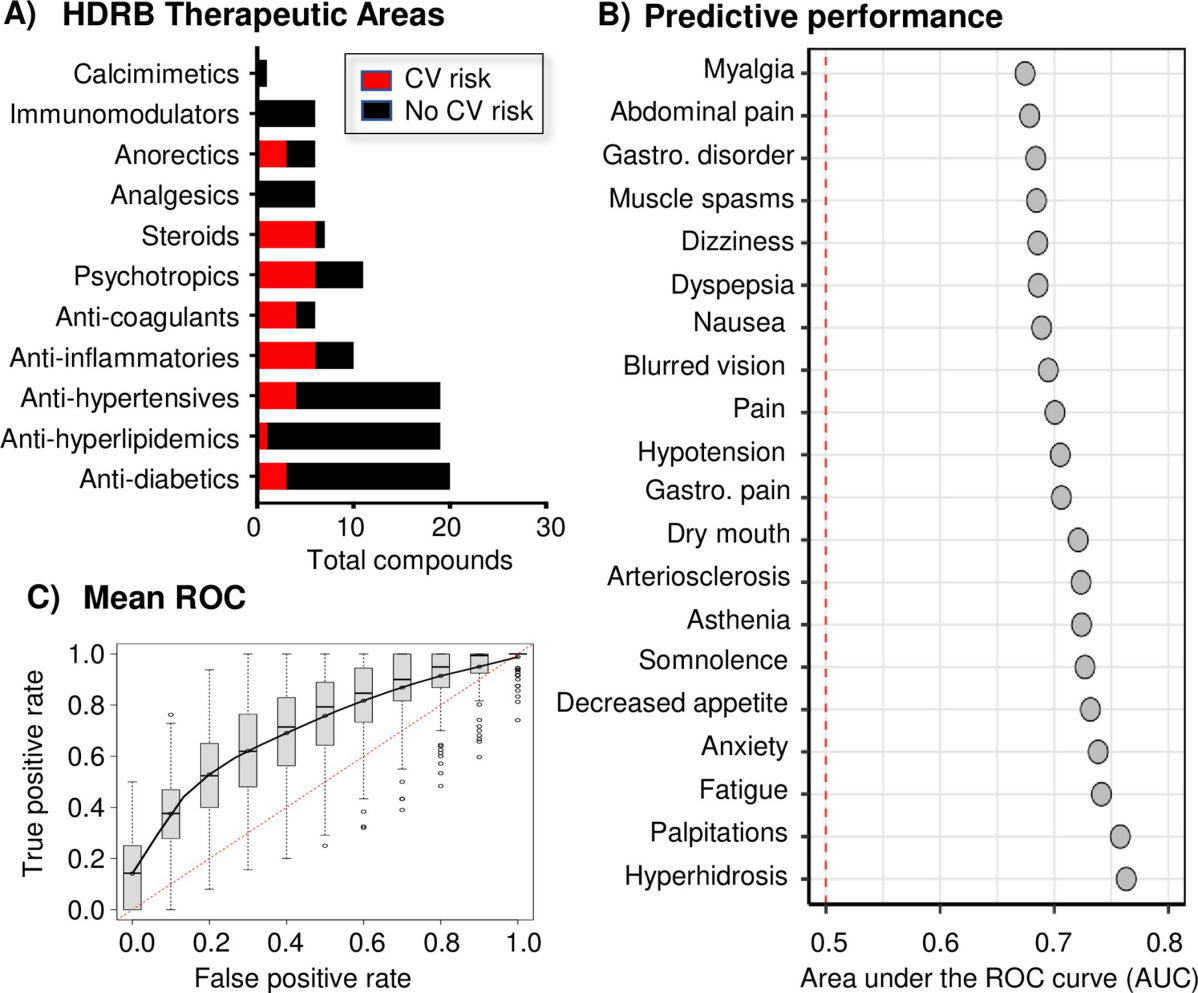
Human response database. **A)** Compounds tested in the vascular model (n = 111) in order to build the database and depicted as drug class histogram with known cardiovascular (CV) risk highlighted**. B)** Predictive performance of our classifiers across many adverse events (AUC = 0.5 is the performance of a random classifier). **C)** The mean receiver operating characteristic (ROC) curve for arteriosclerosis prediction. The distributions represent the performance across a 150 bootstrap cross-validation.

The advanced *in vitro* vascular system aimed to capture key pathological drivers of atherosclerosis. Atherosclerosis is a focal inflammatory disease found in bifurcating or bulbous vessel geometries, such as in the internal carotid sinus, which are characterized by low mean shear stresses and a high oscillatory shear index [18,28]. By comparison, straighter geometries found in the common carotid artery, have higher, unidirectional, pulsatile shear stress and are protected from atherosclerotic disease [18,28,35]. This study utilized a cone-and-plate device to recapitulate MRI-measured hemodynamics from the internal carotid sinus to prime the primary human EC and vascular SMC co-culture (**Fig 2A and 2B**) towards a disease phenotype. Eighteen hours later, tumor necrosis factor-α and oxidized low-density lipoprotein were added to heighten the atheroprone state [16] (**Fig 2C**). Twenty-four hours later, vehicle, or compounds were added to the atheroprone model. Test compounds were incubated for an additional twenty-four hours (forty-eight hours total experiment duration). EC and SMC transcriptomic responses were captured separately within the atheroprone model and compared to a non-disease control that utilized human atheroprotective hemodynamics derived from the common carotid artery, a vessel region that does not develop vascular disease. This experimental paradigm was completed with 111 compounds that span a diverse range of structures, targets and disease indications with the goal of developing a predictive vascular pharmacology model (**Fig 3A**). Of these compounds, 107 are clinical stage and 33 carry FDA cardiovascular risk warnings (**Fig 3A, S1 Table**). Many of the compounds are well studied with known side effect profiles and were selected to aid predictions around potential safety concerns, such as cardiovascular risk. Cardiovascular risk associated with each of the test compounds are varied in target mechanism and no common chemical motif can explain the observed cardiovascular risk. Machine learning classification models were created to determine if the transcriptomic profiles encode information predictive of adverse events. Indeed, the system demonstrated good predictive performance across a diverse set of side effects described in SIDER DB [32] (**Fig 3B**), including arteriosclerosis (**Fig 3C**). Together, these results show that the HRDB contains transcriptomic patterns that correlate with many adverse events, which include cardiovascular risk.

**Fig 4:**
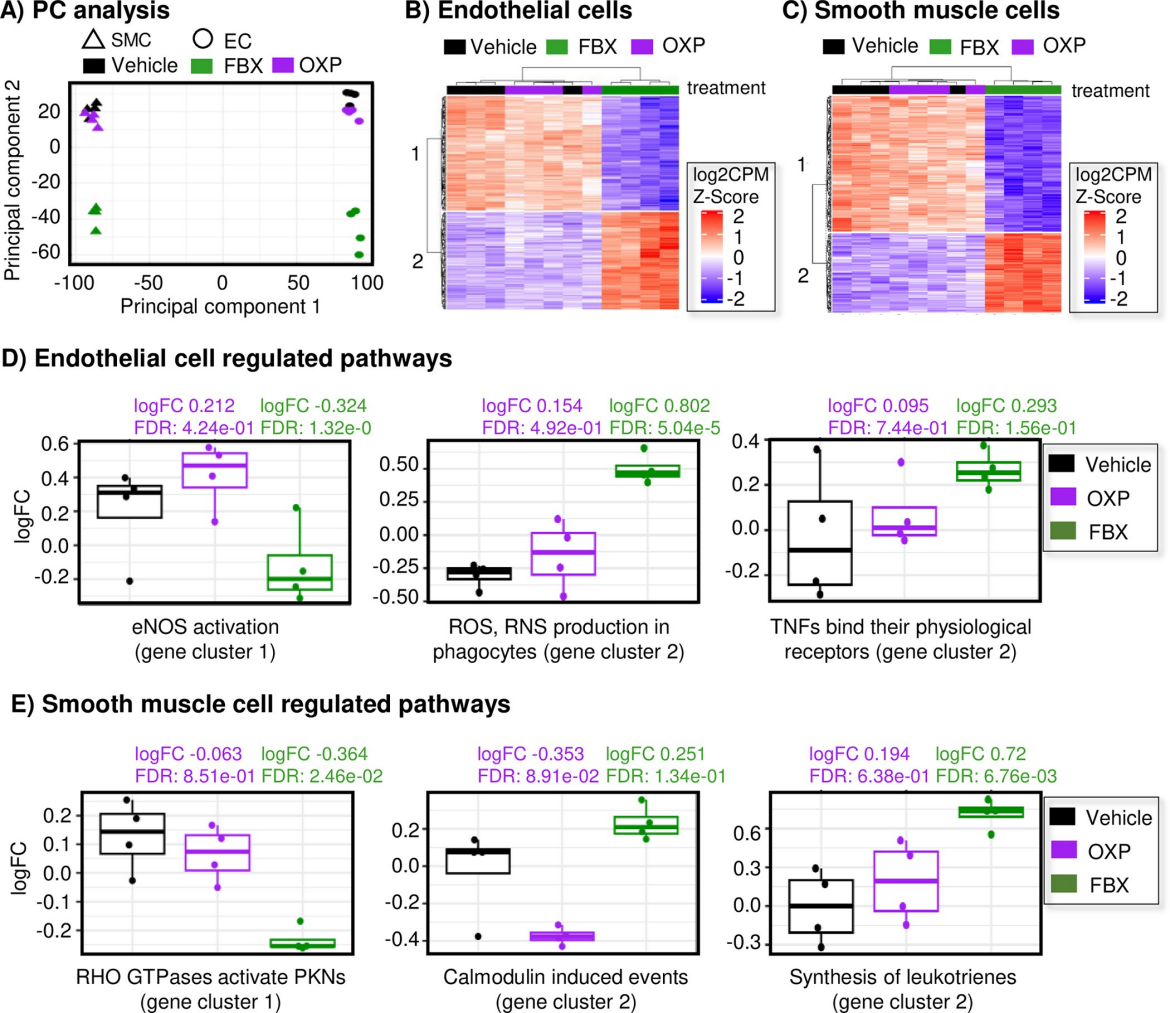
Xanthine oxidase pathway response. **A)** Principle component analysis of DEGs show febuxostat (FBX, 100 μM, green) is different from vehicle (black) and oxypurinol (OXP, 500 μM, red). **B, C)** Heatmaps from PCA; PC1 and PC2 represent 2 different gene clusters. Gene expression (log2CPM Z-score; red = upregulation, blue = downregulation); columns represent treatment replicates; n = 4 in ECs (**B**) or SMCs (**C**). **D, E)** Select pathways regulated by XOi in ECs (**D**) or SMCs (**E**). ECs = endothelial cells, SMCs = smooth muscle cells. LogFC = log fold change, FDR = false discovery rate.

Differential expression and pathway analysis was performed for the transcriptomic response of XOis using the same protocol as the 111 compounds used to construct the HRDB. Vehicle treated ECs and SMCs exhibited a large number of differentially expressed genes (DEGs) (EC: 3,150; SMC: 626; FDR ≤ 10%) in the atheroprone model compared to the atheroprotective control, as expected. Furthermore, amongst the XOis, febuxostat elicited the strongest DEG response in both cell types from the atheroprone model (EC: 2,250; SMC: 3,380; FDR ≤ 10%) relative to the vehicle control. The allopurinol active metabolite, oxypurinol, elicited only a modest response. Principle component analysis revealed febuxostat clearly separated from oxypurinol and the vehicle control in atheroprone ECs and SMCs, demonstrating these XOis elicit transcriptionally distinct biologic responses (**Fig 4A**). These differences were evident in the DEG cluster heatmaps (**Fig 4B and 4C**). Subsequent cluster pathway analysis showed febuxostat downregulated nitric oxide (NO) signaling pathway expression concomitant with increased reactive oxygen species (ROS) and inflammatory signaling pathway expression in ECs (**Fig 4D**). Related to the reduced NO signaling expression in ECs, febuxostat-regulated SMC pathways were associated with contractility (e.g., calmodulin and Rho GTPase) (**Fig 4E**). Expression of pathways with strong inflammatory effects on vascular cells [36] (e.g., ROS, TNF, and leukotriene pathways) were elevated by febuxostat relative to vehicle or oxypurinol in both cell types (**Fig 4D and 4E**). Collectively, febuxostat, but not oxypurinol, modulation of these pathways may be indictive of deleterious effects of febuxostat under atheroprone conditions [37,38].

**Fig 5:**
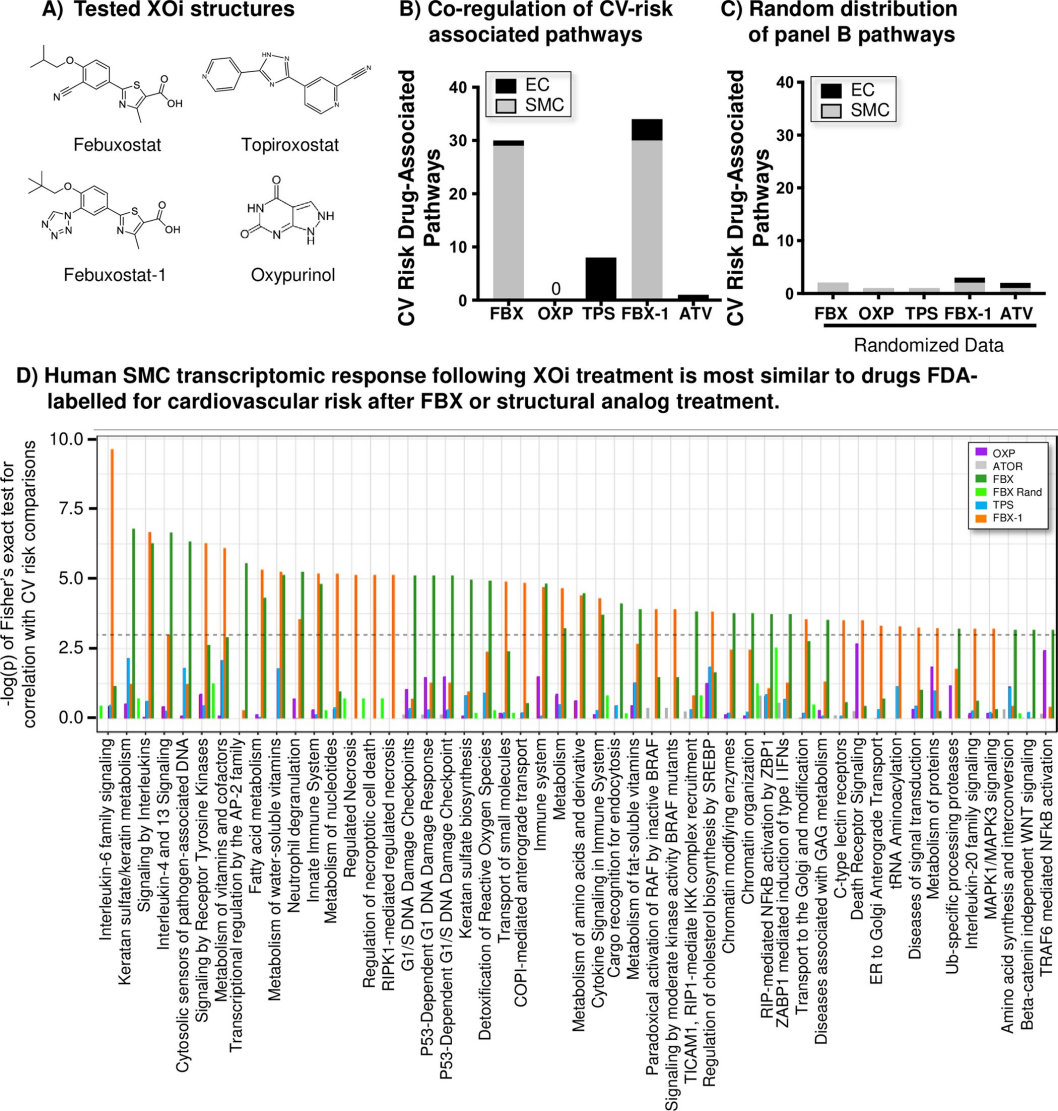
Febuxostat and other drugs labeled for cardiovascular risk regulate similar signaling pathways. **A)** Structures of febuxostat, oxypurinol, topiroxostat, and febuxostat-1. **B)** The total number of pathways regulated by drugs with known cardiovascular risk that are also regulated by febuxostat (FBX), febuxostat-1 (FBX-1), topiroxostat (TPS), oxypurinol (OXP), or atorvastatin (ATV). **C)** In a control analysis, the magnitude of transcripts modulated in panel B) were randomly distributed and analyzed for overlap with pathways regulated by drugs with known cardiovascular risk. **D)** Transcriptomic response of XOi-treated SMCs positively correlated to compounds FDA-labelled for increased cardiovascular risk. The p-value (X-axis) is impacted by the number of drugs regulating the same pathway. Significant association, defined as p < 0.05 (dotted line), plotted as -log(P). XOis evaluated: Febuxostat (100 μM), febuxostat-1 (100 μM), topiroxostat (100 μM), oxypurinol (100 μM), and controls atorvastatin (497 nM), and febuxostat rand (randomized gene names).

Next, utility of the HRDB to detect cardiovascular risk was tested for several xanthine oxidase inhibitors (**Fig 5A**). The dataset underwent statistical analysis for quantification of shared pathways regulated by compounds with known cardiovascular risk. In SMCs, the febuxostat transcriptomic response strongly correlated with 29 pathways regulated in common by drugs FDA-labelled for increased cardiovascular risk (**Fig 5B and 5D**). This correlation was not driven solely by higher febuxostat-mediated DEG response, as atorvastatin (a cholesterol-lowering drug with no known cardiovascular risk) exhibited a strong DEG response (EC: 3,245; SMC: 746, FDR ≤ 10%), but did not correlate with cardiovascular risk-associated pathways (**Fig 5B and 5D**), in keeping with its clinical findings. Interestingly, no correlation was observed following oxypurinol treatment in either cell type (**Fig 5B and 5D**), and heightened cardiovascular risk is not associated with this compound. As additional controls, no correlation was observed following randomization of the febuxostat-regulated transcript response (**Fig 5C**) and observed correlations in the SMCs are greater than expected by chance. Lastly, minimal overlap in pathways modulated by drugs labelled for increased cardiovascular risk was observed in the ECs for each of the XOis evaluated (**Fig 5B**).

Because febuxostat is structurally unrelated to purine-based allopurinol or oxypurinol, we hypothesized that the observed cardiovascular risk stems from chemical structure. To test this hypothesis, two additional non-purine compounds were tested under atheroprone conditions: a febuxostat structural analog (US patent 9,815,826 B2), labelled febuxostat-1, and topiroxostat, a structurally distinct compound (**Fig 5A**). Similar to febuxostat, febuxostat-1 strongly correlated with cardiovascular risk-associated pathways in SMCs while topiroxostat showed no correlation (**Fig 5B and 5D**). Pairwise comparison of febuxostat and febuxostat-1 indicated strong DEG overlap, which was not observed with any other compound. As above, neither febuxostat-1 nor topiroxostat showed association with cardiovascular risk in ECs (**Fig 5B and 5D**). Together, transcriptional profiling in this advanced *in vitro* model suggests that differences in cardiovascular risk can be detected pre-clinically and that these data support the FDA cardiovascular warning in individuals with cardiovascular risk for febuxostat, while echoing the safety profile clinically observed with allopurinol and topiroxostat.

## Discussion

These data demonstrate the utility of advanced *in vitro* models to provide additional mechanistic insight into the safety and signaling pathways modulated by potential new therapeutics. By comparing transcriptomic responses from xanthine oxidase inhibitors to over 100 diverse compounds within a physiologically relevant human model that incorporates multiple human cell types with tissue architecture, perfusion, and hemodynamics, we were able to demonstrate febuxostat, and its chemical analog, regulates gene expression and signaling pathways similar to several other drugs FDA-labelled for cardiovascular risk. Moreover, the febuxostat mechanism underlying CV risk pathways was distinct from oxypurinol and topiroxostat that are not associated with cardiovascular risk despite also targeting xanthine oxidase.

These data are timely given that the febuxostat FDA warning was recently challenged by the FAST clinical trial (EudraCT 2011-001883-23) [26]. In contrast, our data support the CARES trial where febuxostat increased cardiovascular-related death incidence in atheroprone individuals (NCT01101035) [25]. Importantly, because the FAST trial excluded patients with cardiovascular disease while allowing co-treatment of colchicine (a strong anti-inflammatory), our data do not speak directly to FAST findings, but our data do continue to support FDA warnings motivated by the CARES trial.

This study also extends earlier work examining vascular xanthine oxidase [39]. Here, febuxostat and its analog febuxostat-1 regulated EC inflammatory, oxidative stress, and nitric oxide pathways concomitantly with SMC contractility pathways. Moreover, the pattern of pathway regulation was similar to that mediated by other drugs FDA-labeled for cardiovascular risk. Other, structurally dissimilar XOis, (i.e., oxypurinol and topiroxostat), did not regulate these pathways. Together, these data suggest febuxostat-associated cardiovascular risk may stem from inhibitor chemical structure rather than xanthine oxidase inhibition, itself. In partial support, subjects lacking functional xanthine oxidase are largely asymptomatic [40], and a large patient population is maintained on other XOis, such as allopurinol [41–43].

Our study has several limitations. Many mechanisms likely contribute to cardiovascular risk, and the role of chemical motifs or causal pathways for the 33 test articles carrying FDA-labeled cardiovascular risk remain unknown. Thus, we took an unbiased approach to determine if cardiovascular signaling pathways regulated by febuxostat may overlap with pathways modulated by drugs with known cardiovascular risk. Regardless, it is critical that future work interrogate pathways we identified to evaluate their correlation to cardiovascular risk. Additionally, compounds were only evaluated in the context of an atheroprone environment, which is more similar to the CARES rather than the FAST trial; however, these data suggest this platform should be used towards that end in future work. Despite these limitations, we suggest these data both support CARES while providing an excellent platform to better understand both FAST trial outcomes and novel medications under development in future work.

In summary, comparing the transcriptomic response of novel drug entities within advanced human disease model systems could provide insight into potential liabilities that may not otherwise be uncovered until large and late-stage clinical trials are conducted. This potential utility is demonstrated here using the FAST-CARES trial inspired controversy over FDA cardiovascular risk labelling of the gout medication febuxostat. Due to febuxostat-mediated signaling within human SMCs in a pattern similar to other drugs FDA-labeled for heightened cardiovascular risk, this model supports the FDA warning and further suggests that this liability may be related to chemical structure rather than on-target activity. Results of this study also support the promise of developing and utilizing new drug discovery tools to uncover potential risk earlier in the drug development process and could help enable best-in-class and first-in-class future therapeutics.

## Supporting information

S1 Table Compounds that compose the Human-Response Database (HRDB). 111 compounds alone or in combination were utilized to create the HRDB, of which many of these have known cardiovascular risk. Abbreviations: MI: Myocardial infarction; GI: Gastrointestinal; OTC: Over-the-counter.
(XLSX)

## References

[1] Harrison RK. Phase II and phase III failures: 2013–2015. Nat Rev Drug Discov. 2016;15: 817–818. doi: 10.1038/nrd.2016.184 27811931

[2] Sun D, Gao W, Hu H, Zhou S. Why 90% of clinical drug development fails and how to improve it? Acta Pharm Sin B. 2022;12: 3049–3062. doi: 10.1016/j.apsb.2022.02.002 35865092 PMC9293739

[3] Armstrong AJ, Collado MS, Henke BR, Olson MW, Hoang SA, Hamilton CA, et al. A novel small molecule approach for the treatment of propionic and methylmalonic acidemias. Mol Genet Metab. 2021;133: 71–82. doi: 10.1016/j.ymgme.2021.03.001 33741272 PMC9109253

[4] Armstrong AJ, Henke BR, Collado MS, Taylor JM, Pourtaheri TD, Dillberger JE, et al. Identification of 2,2-Dimethylbutanoic Acid (HST5040), a Clinical Development Candidate for the Treatment of Propionic Acidemia and Methylmalonic Acidemia. J Med Chem. 2021;64: 5037–5048. doi: 10.1021/acs.jmedchem.1c00124 33848153

[5] Chapman KA, Collado MS, Figler RA, Hoang SA, Armstrong AJ, Cui W, et al. Recapitulation of metabolic defects in a model of propionic acidemia using patient-derived primary hepatocytes. Mol Genet Metab. 2016;117: 355–362. doi: 10.1016/j.ymgme.2015.12.008 26740382 PMC4852394

[6] Collado MS, Armstrong AJ, Olson M, Hoang SA, Day N, Summar M, et al. Biochemical and anaplerotic applications of in vitro models of propionic acidemia and methylmalonic acidemia using patient-derived primary hepatocytes. Mol Genet Metab. 2020;130: 183–196. doi: 10.1016/j.ymgme.2020.05.003 32451238 PMC7337260

[7] Roller DG, Hoang SA, Rawls KD, Owen KA, Simmers MB, Figler RA, et al. Validation of a multicellular tumor microenvironment system for modeling patient tumor biology and drug response. Sci Rep. 2021;11: 5535. doi: 10.1038/s41598-021-84612-z 33692370 PMC7946945

[8] Dash A, Simmers MB, Deering TG, Berry DJ, Feaver RE, Hastings NE, et al. Hemodynamic flow improves rat hepatocyte morphology, function, and metabolic activity in vitro. Am J Physiol Cell Physiol. 2013;304: C1053–63. doi: 10.1152/ajpcell.00331.2012 23485712 PMC3677175

[9] Terelius Y, Figler RA, Marukian S, Collado MS, Lawson MJ, Mackey AJ, et al. Transcriptional profiling suggests that Nevirapine and Ritonavir cause drug induced liver injury through distinct mechanisms in primary human hepatocytes. Chem Biol Interact. 2016;255: 31–44. doi: 10.1016/j.cbi.2015.11.023 26626330 PMC4889565

[10] Yu S, Ericson M, Fanjul A, Erion DM, Paraskevopoulou M, Smith EN, et al. Genome-wide CRISPR Screening to Identify Drivers of TGF-β-Induced Liver Fibrosis in Human Hepatic Stellate Cells. ACS Chem Biol. 2022;17: 918–929. doi: 10.1021/acschembio.2c00006 35274923 PMC9016707

[11] Feaver RE, Cole BK, Lawson MJ, Hoang SA, Marukian S, Blackman BR, et al. Development of an in vitro human liver system for interrogating nonalcoholic steatohepatitis. JCI Insight. 2016;1: e90954. doi: 10.1172/jci.insight.90954 27942596 PMC5135271

[12] Dash A, Figler RA, Blackman BR, Marukian S, Collado MS, Lawson MJ, et al. Pharmacotoxicology of clinically-relevant concentrations of obeticholic acid in an organotypic human hepatocyte system. Toxicol In Vitro. 2017;39: 93–103. doi: 10.1016/j.tiv.2016.11.014 27939613 PMC5191970

[13] Shepherd EL, Saborano R, Northall E, Matsuda K, Ogino H, Yashiro H, et al. Ketohexokinase inhibition improves NASH by reducing fructose-induced steatosis and fibrogenesis. JHEP Rep. 2021;3: 100217. doi: 10.1016/j.jhepr.2020.100217 33490936 PMC7807164

[14] Simmers MB, Cole BK, Ogletree ML, Chen Z, Xu Y, Kong L-J, et al. Hemodynamics associated with atrial fibrillation directly alters thrombotic potential of endothelial cells. Thromb Res. 2016;143: 34–9. doi: 10.1016/j.thromres.2016.04.022 27179130

[15] Collado MS, Cole BK, Figler RA, Lawson M, Manka D, Simmers MB, et al. Exposure of Induced Pluripotent Stem Cell-Derived Vascular Endothelial and Smooth Muscle Cells in Coculture to Hemodynamics Induces Primary Vascular Cell-Like Phenotypes. Stem Cells Transl Med. 2017;6: 1673–1683. doi: 10.1002/sctm.17-0004 28628273 PMC5689791

[16] Cole BK, Simmers MB, Feaver R, Qualls CW, Collado MS, Berzin E, et al. An In Vitro Cynomolgus Vascular Surrogate System for Preclinical Drug Assessment and Human Translation. Arterioscler Thromb Vasc Biol. 2015;35: 2185–95. doi: 10.1161/ATVBAHA.115.306245 26293464

[17] Hastings NE, Simmers MB, McDonald OG, Wamhoff BR, Blackman BR. Atherosclerosis-prone hemodynamics differentially regulates endothelial and smooth muscle cell phenotypes and promotes pro-inflammatory priming. Am J Physiol Cell Physiol. 2007;293: C1824–33. doi: 10.1152/ajpcell.00385.2007 17913848

[18] Feaver RE, Gelfand BD, Blackman BR. Human haemodynamic frequency harmonics regulate the inflammatory phenotype of vascular endothelial cells. Nat Commun. 2013;4: 1525. doi: 10.1038/ncomms2530 23443553 PMC4100071

[19] Feaver RE, Hastings NE, Pryor A, Blackman BR. GRP78 upregulation by atheroprone shear stress via p38-, alpha2beta1-dependent mechanism in endothelial cells. Arterioscler Thromb Vasc Biol. 2008;28: 1534–41. doi: 10.1161/ATVBAHA.108.167999 18556570 PMC2723835

[20] Feaver RE, Gelfand BD, Wang C, Schwartz MA, Blackman BR. Atheroprone hemodynamics regulate fibronectin deposition to create positive feedback that sustains endothelial inflammation. Circ Res. 2010;106: 1703–11. doi: 10.1161/CIRCRESAHA.109.216283 20378855 PMC2891748

[21] Orr AW, Stockton R, Simmers MB, Sanders JM, Sarembock IJ, Blackman BR, et al. Matrix-specific p21-activated kinase activation regulates vascular permeability in atherogenesis. J Cell Biol. 2007;176: 719–27. doi: 10.1083/jcb.200609008 17312022 PMC2064028

[22] FitzGerald JD, Dalbeth N, Mikuls T, Brignardello-Petersen R, Guyatt G, Abeles AM, et al. 2020 American College of Rheumatology Guideline for the Management of Gout. Arthritis Care Res (Hoboken). 2020;72. doi: 10.1002/acr.24180 32391934 PMC10563586

[23] Abdellatif AA, Elkhalili N. Management of gouty arthritis in patients with chronic kidney disease. Am J Ther. 2014;21. doi: 10.1097/MJT.0b013e318250f83d 22960848

[24] Krishnan E. Reduced Glomerular Function and Prevalence of Gout: NHANES 2009–10. PLoS One. 2012;7. doi: 10.1371/journal.pone.0050046 23209642 PMC3507834

[25] White WB, Saag KG, Becker MA, Borer JS, Gorelick PB, Whelton A, et al. Cardiovascular Safety of Febuxostat or Allopurinol in Patients with Gout. New England Journal of Medicine. 2018. doi: 10.1056/nejmoa1710895 29527974

[26] Choi HK, Neogi T, Stamp LK, Terkeltaub R, Dalbeth N. Reassessing the Cardiovascular Safety of Febuxostat: Implications of the Febuxostat versus Allopurinol Streamlined Trial. Arthritis and Rheumatology. 2021;73. doi: 10.1002/art.41638 33403821 PMC10520949

[27] Mackenzie IS, Ford I, Nuki G, Hallas J, Hawkey CJ, Webster J, et al. Long-term cardiovascular safety of febuxostat compared with allopurinol in patients with gout (FAST): a multicentre, prospective, randomised, open-label, non-inferiority trial. The Lancet. 2020;396. doi: 10.1016/S0140-6736(20)32234-0 33181081

[28] Gelfand BD, Epstein FH, Blackman BR. Spatial and spectral heterogeneity of time-varying shear stress profiles in the carotid bifurcation by phase-contrast MRI. Journal of Magnetic Resonance Imaging. 2006;24. doi: 10.1002/jmri.20765 17083089

[29] Patro R, Duggal G, Love MI, Irizarry RA, Kingsford C. Salmon provides fast and bias-aware quantification of transcript expression. Nat Methods. 2017;14. doi: 10.1038/nmeth.4197 28263959 PMC5600148

[30] Soneson C, Love MI, Robinson MD. Differential analyses for RNA-seq: transcript-level estimates improve gene-level inferences. F1000Res. 2015;4. doi: 10.12688/f1000research.7563.2 26925227 PMC4712774

[31] Robinson MD, McCarthy DJ, Smyth GK. edgeR: A Bioconductor package for differential expression analysis of digital gene expression data. Bioinformatics. 2009;26. doi: 10.1093/bioinformatics/btp616 19910308 PMC2796818

[32] Kuhn M, Letunic I, Jensen LJ, Bork P. The SIDER database of drugs and side effects. Nucleic Acids Res. 2016;44: D1075–9. doi: 10.1093/nar/gkv1075 26481350 PMC4702794

[33] Tatonetti NP, Ye PP, Daneshjou R, Altman RB. Data-driven prediction of drug effects and interactions. Sci Transl Med. 2012;4: 125ra31. doi: 10.1126/scitranslmed.3003377 22422992 PMC3382018

[34] Kuhn Max. Building Predictive Models in R Using the caret Package. J Stat Softw. 2008; 1–26.

[35] DeBakey ME, Lawrie GM, Glaeser DH. Patterns of atherosclerosis and their surgical significance. Ann Surg. 1985;201: 115–31. doi: 10.1097/00000658-198502000-00001 3155934 PMC1250631

[36] Wasserman AH, Venkatesan M, Aguirre A. Bioactive Lipid Signaling in Cardiovascular Disease, Development, and Regeneration. Cells. 2020. doi: 10.3390/cells9061391 32503253 PMC7349721

[37] Basatemur GL, Jørgensen HF, Clarke MCH, Bennett MR, Mallat Z. Vascular smooth muscle cells in atherosclerosis. Nature Reviews Cardiology. 2019. doi: 10.1038/s41569-019-0227-9 31243391

[38] Bennett MR, Sinha S, Owens GK. Vascular Smooth Muscle Cells in Atherosclerosis. Circ Res. 2016;118. doi: 10.1161/CIRCRESAHA.115.306361 26892967 PMC4762053

[39] Malik UZ, Hundley NJ, Romero G, Radi R, Freeman BA, Tarpey MM, et al. Febuxostat inhibition of endothelial-bound XO: Implications for targeting vascular ROS production. Free Radic Biol Med. 2011;51. doi: 10.1016/j.freeradbiomed.2011.04.004 21554948 PMC3130629

[40] Akıncı N, Cakıl A, Oner A. Classical xanthinuria: a rare cause of pediatric urolithiasis. Türk Üroloji Dergisi/Turkish Journal of Urology. 2013;39. doi: 10.5152/tud.2013.066 26328123 PMC4548614

[41] Meyer L, Ford C, Harrison K, Ghuman NK, Matlin OS, Palmieri A, et al. Trends in Medication Utilization and the Cost of Treatment for Gout. Available: www.ajpblive.com.

[42] Kim SC, Neogi T, Kim E, Lii J, Desai RJ. Trends in Utilization of Urate-Lowering Therapies Following the US Food and Drug Administration’s Boxed Warning on Febuxostat. Arthritis and Rheumatology. John Wiley and Sons Inc; 2021. pp. 542–543. doi: 10.1002/art.41550 PMC7914144 33029931

[43] Chen-Xu M, Yokose C, Rai SK, Pillinger MH, Choi HK. Contemporary Prevalence of Gout and Hyperuricemia in the United States and Decadal Trends: The National Health and Nutrition Examination Survey, 2007–2016. Arthritis and Rheumatology. 2019;71: 991–999. doi: 10.1002/art.40807 30618180 PMC6536335

